# Conducting descriptive epidemiology and causal inference studies using observational data: A 10-point primer for stroke researchers

**DOI:** 10.1177/23969873251332118

**Published:** 2025-04-19

**Authors:** Leonid Churilov, Kathryn Hayward, Vignan Yogendrakumar, Nadine Andrew

**Affiliations:** 1 Department of Medicine (Royal Melbourne Hospital), University of Melbourne, Heidelberg, VIC, Australia; 2 Department of Neurology, Royal Melbourne Hospital, Parkville, Australia; 3 Melbourne School of Health Sciences, University of Melbourne, Heidelberg, VIC, Australia; 4 Division of Neurology, The Ottawa Hospital and Ottawa Hospital Research Institute, University of Ottawa, Ottawa, ON, Canada; 5 Department of Medicine, Peninsula Clinical School, Central Clinical School, Monash University, Frankston, VIC, Australia; 6 National Centre for Healthy Ageing, Frankston, VIC, Australia

**Keywords:** Stroke, causal inference, observational data

## Abstract

Routinely-collected health data and emerging data-linkage capabilities provide researchers and clinicians with rich opportunities to answer important research questions by conducting observational studies. We provide stroke researchers with 10 important points to consider and implement to ensure the validity and interpretability of descriptive epidemiology and causal inference studies based on observational data. We discuss different types of observational studies and biases that may arise in such studies. We review types of causal effects and the use of Target Trial emulation and Directed Acyclic Graphs to improve validity of observational studies. We also illustrate appropriate and inappropriate use of covariate adjustment for the analyses of observational studies and review the methods for estimating the effects of treatments, interventions, and exposures in causal inference studies. Finally, we provide recommendations for clinical researchers and journal manuscript reviewers in stroke domain and beyond for the appropriate use and reporting of these methods.

## Point 1: Consider the type of data you will use: data collected specifically for research versus health data collected for administrative and clinical purposes (but not specific research goals)

While randomized clinical trials (RCTs) remain the main source of evidence to inform clinical practice, access to clinical registries, electronic medical records, data capture systems and linkage capabilities has prompted clinicians and researchers to try to answer research questions using routinely-collected observational health data. Such data are defined in The Reporting of studies Conducted using Observational Routinely-collected health Data (RECORD) Statement guideline as “routinely collected health data, obtained for administrative and clinical purposes without specific a priori research goals.”^1^

This is an efficient use of resource but there are key differences between a traditional cohort or cross-sectional observational study with prospective data collection versus a research study that uses routinely collected health data. As these data are usually routinely collected for operational and clinical purposes, there are challenges in their application to clinical research. While the basic goal of using a sample to estimate an endpoint of interest in order to generalize the findings back to that population remains the same, the practical steps to achieve that goal, and – more important – the implications of including or excluding certain data, differ substantially.

With a prospective study, a research question is first formulated and the population on whom the answers will apply should be defined. Next, the approach to collecting the study sample is planned (e.g. “we aim to recruit every patient attending a particular set of stroke clinics”). The relevant data are then collected. Once the decision is taken about which data to collect, missing data are deeply undesirable. At best, if the data are missing completely at random, we risk a falsely neutral study result (i.e. a type II error). If we are less fortunate, and there is a systematic pattern to the data missingness, a bias is introduced. Then, even complex statistical techniques may not necessarily restore study validity.

The situation is different when a research study uses routinely collected health data. Here, inevitably there will be redundant data, that are not relevant to the research question. In particular, it may be critical to exclude data from patients who lie beyond the population of interest, otherwise misleading conclusions may be drawn. For example, consider that routine stroke admissions data will include ischemic stroke, haemorrhagic stroke, TIA and stroke mimics: a question directed at reperfusion strategies would not be reliably answered on the unselected population.

RECORD guidance supplements the STROBE statement^2,3^ to assist reporting of observational studies that use routinely collected health data. Among the many RECORD recommendations, the most relevant for this discussion are those designed to minimize potential selection bias that may undermine the generalizability of the study findings, and those that ensure valid use of opportunistic data.

**Figure 1.**
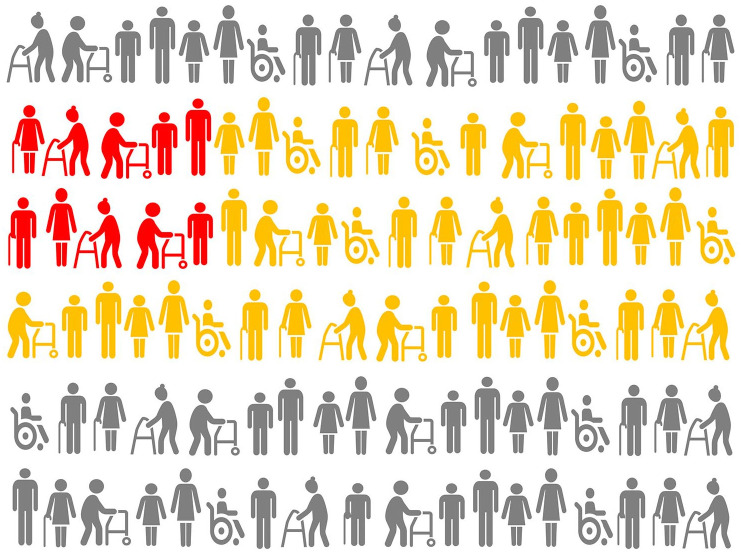
Illustrating Point 1. Hierarchy of populations by RECORD Guidelines: source population is shown in gray, database population is shown in yellow, study population is shown in red. Note that the study population may not necessarily fully and appropriately reflect the source population and it is important to examine whether database population is adequate for the study population to be generalized to the intended source population in order to answer the research question(s).

RECORD guidelines propose the following hierarchy of populations as illustrated in Figure 1:

- The *source population*: the population the researchers want to study and generalize their findings to;
- The *database population*: derived from the source population and reflects people with data included in the data source;
- The *study population*: a subset of the database population identified by the researchers using codes and algorithms.

We can illustrate this with the International Stroke Perfusion Imaging Registry (INSPIRE). INSPIRE is a web-based data repository collecting imaging and clinical stroke data to measure implementation of advanced imaging in stroke from comprehensive stroke centers across Australia, China, and Canada. Since 2012, it has been recruiting patients with stroke who had acute CTP and CT angiography and follow-up imaging and clinical data. Garcia-Esperon et al^4^ used data from INSPIRE to evaluate the association of endovascular thrombectomy (EVT) with functional outcome in patients with a baseline ischemic core volume > 70 ml. The hierarchy of their study populations can be defined as follows:

- the *source population:* people with ischemic stroke core volume > 70 ml;
- the *database population:* patients with acute ischemic stroke, who had baseline multimodal CT performed within 24 h from stroke onset (including noncontrast CT [NCCT], CTP, and CT angiography (CTA)), from Australia, China, and Canada, and who were prospectively enrolled into the INSPIRE from 2012 to December 2020;
- the *study population:* patients from the database population who had baseline CTP performed within 24 h of last seen well, had baseline CTP ischemic core volume ⩾ 70 ml, and had available 3-month modified Rankin Scale (mRS) scores.

Several RECORD guidelines specifically concern the validity of study population selection and provide useful guidance here. Specifically, RECORD Item 12.1 requires the authors to “describe the extent to which the investigators had access to the database population used to create the study population”; RECORD Item 13.1 guides the authors to “Describe in detail the selection of the persons included in the study (i.e. study population selection) including filtering based on data quality, data availability and linkage” either in the text or by means of a study flow diagram; while RECORD Item 19.1 requires the discussion of the implications of using data that were not created or collected to answer the specific research question(s), and discussion of “misclassification bias, unmeasured confounding, missing data, and changing eligibility over time, as they pertain to the study being reported.”

In summary, studies that use routinely collected, “opportunistic” health data will share standard design and validity concerns with studies that use prospective data collection but are also susceptible to additional sources of bias. These threats to validity need to be managed depending on the nature of the research question as discussed in the subsequent Points below.

### Recommendation 1.1

The RECORD statement should be used as an extension to STROBE guidelines when reporting studies conducted using observational routinely-collected health data.

### Recommendation 1.2

Ask whether the *database population* is adequate for your *study population* to be generalized to the *source population* and thus to answer your research question(s), that is, does your opportunistic dataset have the necessary breadth, depth, and quality of information to let you create a reliable analysis dataset, and if so, can you generalize from that analysis to a well-defined, future patient group?

## Point 2: Consider the research question you are asking: Is it about causality or descriptive epidemiology?

The choice of study design for an observational study is heavily influenced by the research aim and the nature of the questions being asked. A cohesive link between the research aim/questions and the study design/analysis is essential to study validity. However, despite numerous high-quality discussions of these concepts in the epidemiological literature,^5–9^ very few observational studies published in the stroke field explicitly articulate them.

To achieve a valid result from observational studies, we must differentiate among *descriptive, causal*, and *predictive* epidemiology research questions.^5^ Predictive epidemiology questions are beyond of the scope of this discussion, however. We focus here on descriptive and causal research questions.

Consider an example. After several years of the COVID-19 pandemic, we ask whether the number and severity of infection episodes experienced by an individual may be related to their risk of having a stroke within a defined timeframe. This prompts two distinct research questions, that have contrasting study aims:

- *The descriptive epidemiology/association question:* Do the kind of people who experienced multiple severe instances of COVID-19 infection have a higher risk of a stroke event within a given timeframe?
- *The causal inference question:* Does experiencing multiple severe instances of COVID-19 infection increase the risk of stroke event within a given timeframe?

**Figure 2.**
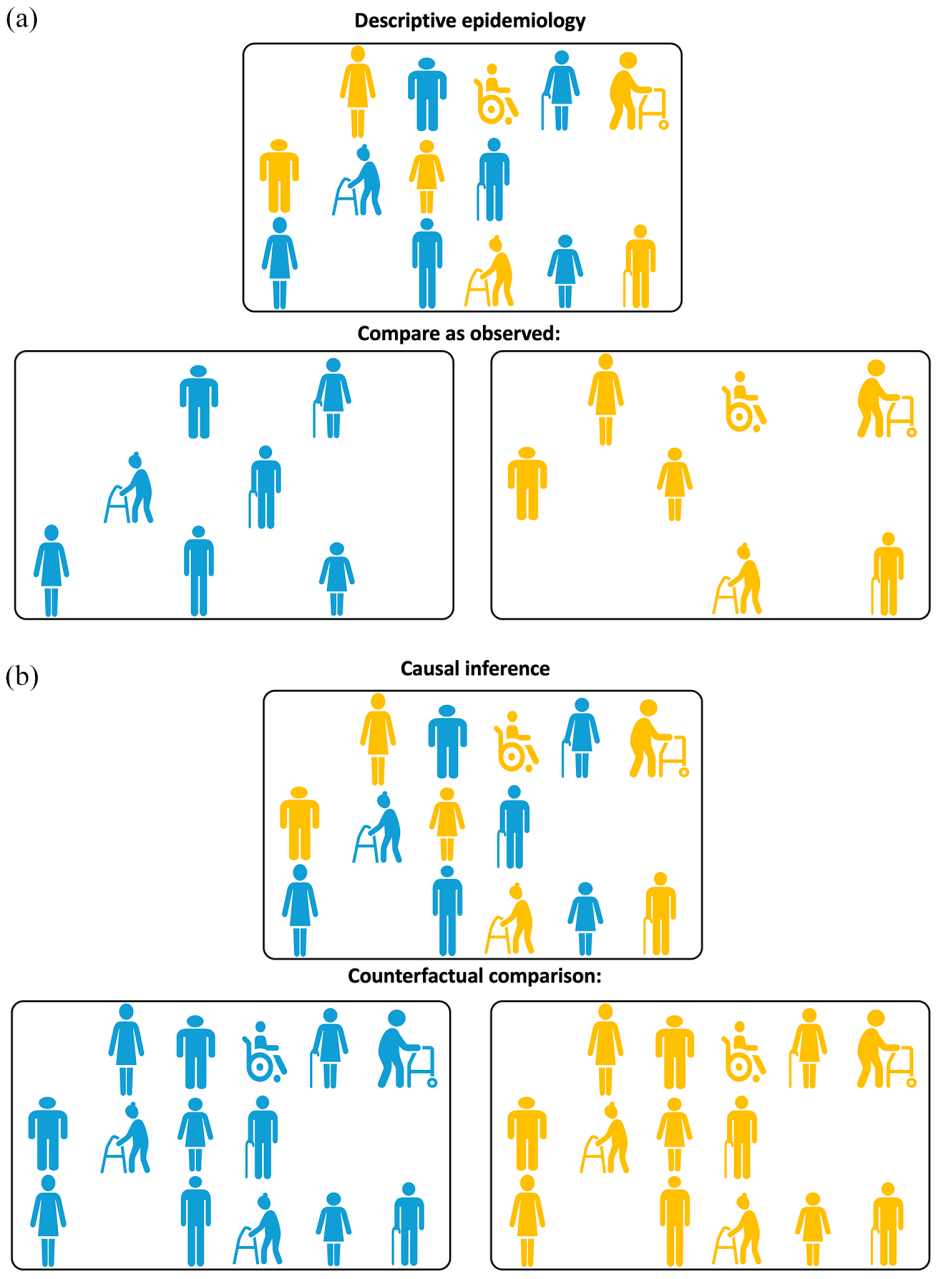
(a) Illustrating Point 2. Comparison “as observed” for descriptive epidemiology questions. To answer a descriptive epidemiology question, unexposed (shown in blue) and exposed (shown in yellow) participants need to be compared as they are observed. (b) Illustrating Point 2. Counterfactual comparison for causal inference questions. To answer a causal inference question, unexposed (shown in blue) and exposed (shown in yellow) participants are compared using counterfactual (i.e. counter to the actual fact of exposure) scenarios: first all the participants, irrespective of the actual exposure received, are assumed to be unexposed (blue) and then all the participants are assumed to be exposed (yellow).

*Descriptive epidemiology/association* studies are typically concerned with “the relationship of disease to basic characteristics such as age, gender, ethnicity, occupation, social class, and geographic location” (p. 72).^10^ Such studies aim to “characterize the distributions of health, disease, and harmful or beneficial exposures in a well-defined population as they exist”, including “any meaningful differences in distribution, and whether that distribution is changing over time” (p. 1175).^5^ To sharpen the emphasis, as illustrated in Figure 2(a), *descriptive* questions focus on the distribution of the outcome in a given population under exposure conditions that the population in fact received. This question is fundamentally different to the related *causal inference* question (Figure 2(b)) that requires consideration of *a hypothetical alternative* (in statistical literature, this is called “counterfactual”) *scenario* where the question asked is *whether the risk of stroke within a given timeframe will be different if people who in reality did not experience multiple severe instances of COVID-19 infection were to experience such exposure*.^11–15^

Why are these two questions so fundamentally different? The main reason is that they require different answers and different analyses. It is possible that the people who experienced multiple severe instances of COVID-19 infection would have a higher risk of stroke even if they were not to experience a COVID-19 infection. This could be because, for example, they may have a higher burden of common stroke risk factors, some of which also predispose them to being infected with COVID-19. Perhaps we have observed a difference in risk of stroke between people with versus without exposure to multiple severe instances of COVID-19 infection. This answer may be perfectly adequate as a *description of what is observed*. At the same time this answer is totally inadequate as a measure of the *causal effect* of COVID-19 exposure on stroke, because it is biased or confounded: it cannot support our reasoning about the alternate scenario *what would have been observed were the unexposed people to end up being exposed*. Examples of various types of research questions relevant to stroke context can be found in Hernan et al.^8^

In summary, while descriptive epidemiology/association questions target study participants under *factual* exposures, causal inference questions are concerned with estimating the outcome of interest under *counterfactual* exposures, i.e. under circumstances that were not actually observed. When asking the causal inference question “Does experiencing multiple severe instances of COVID-19 infection increase the risk of stroke event within a given timeframe,” the researchers would like to gain an understanding of how the exposure (multiple severe COVID-19 infections) changes the outcome (stroke event) for a given population. It does not presume that the population of interest has *actually* been observed under both exposure and non-exposure conditions. In fact, in non-randomized observational studies, it is almost guaranteed that the subpopulation observed to be exposed and the subpopulation observed to be unexposed will be distinct. This is considered further in point 3, below.

### Recommendation 2.1

The descriptive epidemiology versus causal inference nature of the research aim and study questions based on routinely collected observational data needs to be carefully considered and clearly defined in the manuscript. The appropriateness of the study design, chosen analytical approaches, and the validity of resulting answers can only be established in the context of the specified aim.

### Recommendation 2.2

Terminology used in the manuscript needs to reflect the nature of the research aim and questions supported by appropriately conducted study design and analysis. Terms that reflect causal relationships (e.g. “A affects B” or “A impacts B”) should be exclusively reserved for causal inference studies as discussed in Point 3. The term “predict” should be exclusively used in the context of predictive studies. Appropriate phrasing for studies concerned with descriptive epidemiology/association questions would be “A is associated with B.”

## Point 3: Causal inference on observational data

**Figure 3.**
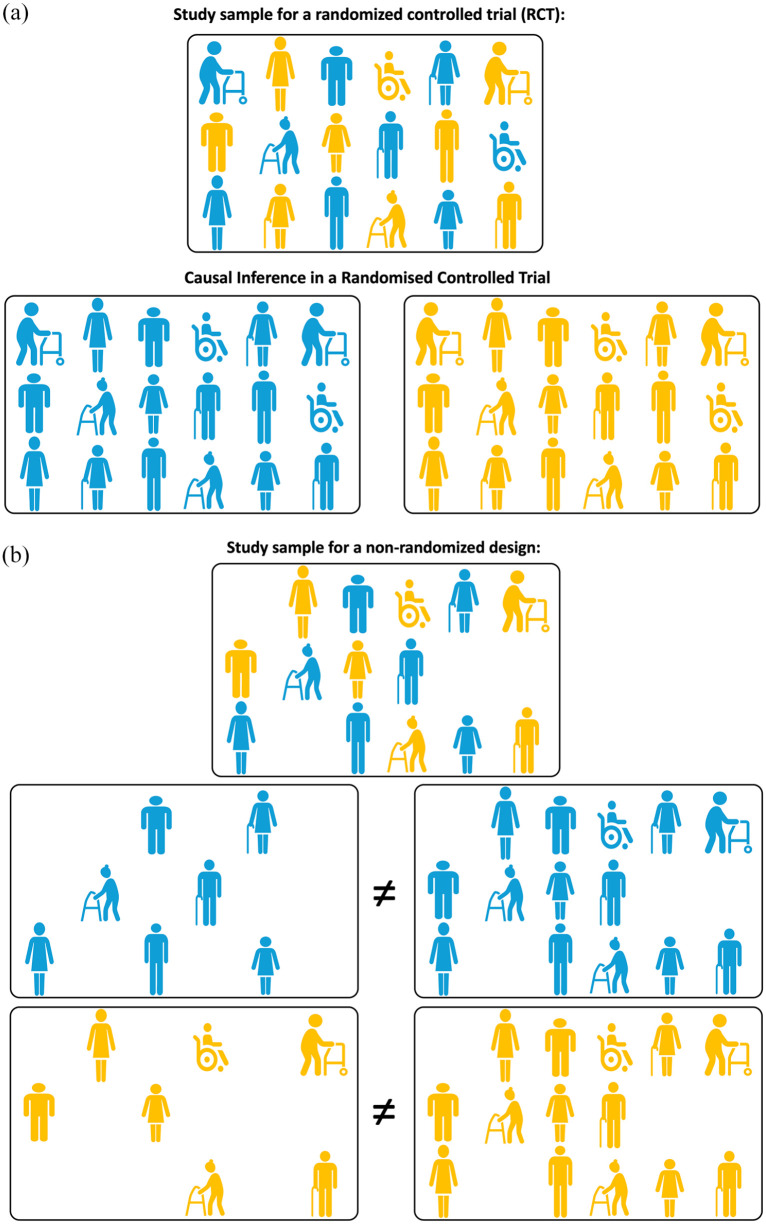
(a) Illustrating Point 3. Causal inference in a randomized control trial (RCT). Due to the randomness of allocation to exposure (treatment), the participants allocated to exposure (treatment, shown in yellow) or control (shown in blue) arm of the trial are assumed to fully and appropriately represent the respective scenarios where the full study sample would have been exposed (treated) or unexposed. (b) Illustrating Point 3. Causal inference in a non-randomized design is subject to confounding. Both unexposed (blue) and exposed (treated, yellow) participants cannot be assumed to fully and appropriately represent scenarios where the full study sample would have been exposed (treated) or unexposed due to potential confounding. Hence, the comparison of unexposed and exposed participants “as observed” cannot provide the answer to a causal inference question.

Randomized clinical trials (RCTs) provide an unbiased estimate of the causal effect of a treatment on an outcome via the mechanism of random assignment. As illustrated in Figure 3(a), due to the randomness of treatment allocation, the participants allocated to the treatment arm or the control arm of the trial are assumed to fully and appropriately represent the respective scenarios where the full study sample would have been treated or untreated respectively, which is essential for answering the causal question as discussed in Point 2.

The focus of the discussion in this and subsequent Points is whether and how causal inference questions can be successfully answered using observational health data. In this point we begin this discussion in the context of Mobile Stroke Units (MSUs) as an illustration. MSUs are custom-built ambulances that enable stroke diagnosis and treatment at the scene, before the patient is taken to a hospital with adequate capacity for the most appropriate treatment (e.g. endovascular thrombectomy). Evaluating the effectiveness of MSUs as a policy intervention requires a comparison of outcomes between the MSU and the existing standard ambulance service stroke response pathways. The most rigorous answer can be obtained by conducting a prospective randomized clinical trial that would directly compare two alternative approaches. If this is not feasible, then instead one may explore routinely collected MSU and standard ambulance data obtained for administrative and clinical purposes.

As discussed in Point 2, at least two fundamentally different questions can be asked:

- A *descriptive epidemiology question* would focus on the relationship of the outcomes of interest to the exposure (the type of pre-hospital stroke care, i.e. MSU vs standard ambulance) and would aim to characterize the distributions of the outcomes and the use of MSU and standard ambulance in a well-defined population, *as they exist*, including any meaningful differences in such distributions. This question would focus on the distributions of the outcome under exposure conditions the population of interest in fact received, that is, both MSU or standard ambulance within the MSU operational radius and solely standard ambulance care outside of this radius.
- A potential *causal inference question* would be whether the outcomes improve in a meaningful way if all the patients were to receive MSU pre-hospital stroke care instead of receiving standard ambulance care.

The first question is straightforward but the second is both more interesting and challenging to answer. Recall that causal inference questions require the consideration of potential counterfactual scenarios. Likely, we will not have observed patients outside of the MSU operational radius to be treated by MSU and we may even have no outcome data on these remote patients. How may we resolve the conflict between the need for counterfactual exposure and the impossibility of arranging this?

The perfect answer to a causal question requires a super-human ability to observe the study participants under two or more mutually exclusive exposure conditions. We ought to observe all the participants receiving MSU treatment and then exactly the same cohort receiving standard ambulance treatment exposures. In reality, we lack the ability to observe the same population under various exposure conditions, but still would like to ask causal questions. The situation is further complicated by the fact that each study participant could be characterized by a number of measured and, possibly, unmeasured *confounders*, that is, variables that may influence both the probability of exposure (e.g. receiving MSU or a standard ambulance treatment: perhaps how well they describe symptoms to prompt dispatch of the MSU) and the outcome of interest (e.g. severity and duration of symptoms). Clinical trials work around this issue with the use of randomization (Figure 3(a)). In most causal inference studies, the aim is to limit the confounding bias.

Rubin’s^11–15^ *potential outcomes framework* for defining causal effects presents a convenient set of tools for thinking about causality, particularly when addressing causal questions on observational data. Under Rubin’s framework, each study participant can have a number of potential outcomes – one outcome for each potential exposure scenario. Taking the MSU study, each patient receiving pre-hospital ambulance stroke care would be assumed to have two potential outcomes: the first if they were to receive the standard ambulance care and the second if they were instead to receive MSU care. According to Rubin’s framework, only one potential outcome can be actually observed for each participant while any other unobserved *counterfactual* potential outcome(s) resulting from unrealized *counterfactual* exposure scenarios is presumed missing. Which specific outcome is observed is determined by the exposure actually experienced by the study participant (Figure 2(b)). In other words, every patient who was treated by standard ambulance has an observed outcome for standard ambulance care but a missing outcome for counterfactual MSU care; and vice versa for every patient who was attended by the MSU.

To use Rubin’s framework for causal reasoning, certain key logical assumptions are needed. First, a *consistency* assumption must be met. It needs to be assumed that were a particular participant to receive a given exposure (e.g. treatment by MSU), their observed outcome would be identical to what was their expected potential outcome for that observed exposure. Second, the participants who actually had a particular exposure status (e.g. treatment by MSU as opposed to the standard ambulance) would have to be representative of what would have occurred had the entire population been given that exposure. This assumption would have allowed the use of the observed data (e.g. outcomes for patients treated by MSU) to reason about the effect of intervening for the entire population (that also includes participants that have not in reality received the intervention). This can be achieved through the appropriate control for confounding. The assumption of no confounding for the effect of exposure on the outcome conditional on covariates is referred to as *exchangeability* or *no unmeasured confounding.* Other important assumptions include *no measurement error*, *correct causal model specification* (discussed in detail in Point 7), and *positivity*^16^ (discussed in detail in Point 9).

Considering the number of assumptions (some of which are difficult to verify) and the central role of “potentially fallible” expert knowledge in causal inference,^8^ any procedure that could satisfy these assumptions and reduce the reliance on the expert causal knowledge is extremely valuable. Randomization is the best-known approach to achieve such goals and to balance potential outcomes at the time of assignment. When a treatment or exposure is randomly assigned, an unbiased estimate of the average causal effect of treatment can be obtained even in the absence of detailed causal knowledge (Figure 3(a)).

In the absence of randomization, the *exchangeability* assumption becomes much harder to verify, as exposure or treatment decisions can depend on both observed and unobserved covariates. This may lead to *confounding*, that is, a situation where a specific condition, illness, or patient characteristic influences both the probability of receiving a particular intervention (exposure) and the outcome of interest (illustrated in Figure 3(b)). *In the absence of randomization, in order to obtain an unbiased causal effect estimate, unverifiable assumptions are inevitable*. These may include an assumption that all baseline covariates that affect treatment or exposure assignment are measured and modeled correctly. Under such assumptions, there are methods for defining causal effect with the *Target Trial* (discussed in detail in Point 6), clear articulation of the causal hypothesis (discussed in detail in Point 7), and deriving unbiased estimates for average causal effects (Point 10).

We must emphasize, though, that methods for causal inference on observational data, despite being designed to alleviate concerns related to the lack of randomization, cannot guarantee that the proper causal inference assumptions are truly met. In particular, if there is reason to suspect that the exchangeability assumption is violated, then causal inference will be misleading. Despite effort made to better understand the impact of potential unmeasured or uncontrolled confounding,^17^ the strength of causal statements made based on observational data is by their very nature lower than from a randomized controlled trial.

### Recommendation 3.1

Recognize that the validity of any causal effect estimate depends on several assumptions that are hard to verify. The strength of causal statements that are based on observational data is therefore lower than for the statements resulting from a randomized controlled trial. Do not proceed if there is reason to suspect violation of an assumption such as exchangeability.

### Recommendation 3.2

Reports of causal studies should clearly list the steps taken to confirm that the relevant causal inference assumptions are met and to alleviate concerns related to the lack of randomization, such as potential for confounding. Rubin’s potential outcomes framework should be used to facilitate clear articulation of exposures, outcomes, and study populations.

## Point 4: Various types of causal effects

Among potential causal inference questions that may be asked about the MSU pre-hospital ambulance stroke treatment case presented in Point 3, consider these three:

- Question 1: What would be the effect if *all the patients were to receive MSU pre-hospital stroke care instead of receiving standard ambulance care*;
- Question 2: What would be the effect of the patients within the *current MSU operational radius receiving MSU pre-hospital stroke care instead of receiving standard ambulance care*;
- Question 3: What would be the effect if the patients outside the current MSU operational radius, who in fact received standard ambulance pre-hospital stroke care, were to receive MSU care?

Note that these differ subtly: they each address a different population. We cannot a priori assume that the sociodemographic, behavioral, clinical, and medical services availability profiles of the patients within the MSU operational radius are comparable to those of the patients residing in more rural or distant zones from the MSU district. We should probably assume that these characteristics will confound the effects of pre-hospital ambulance stroke care on the outcomes of interest and that the potential effects are likely to differ depending on the population in question.

**Figure 4.**
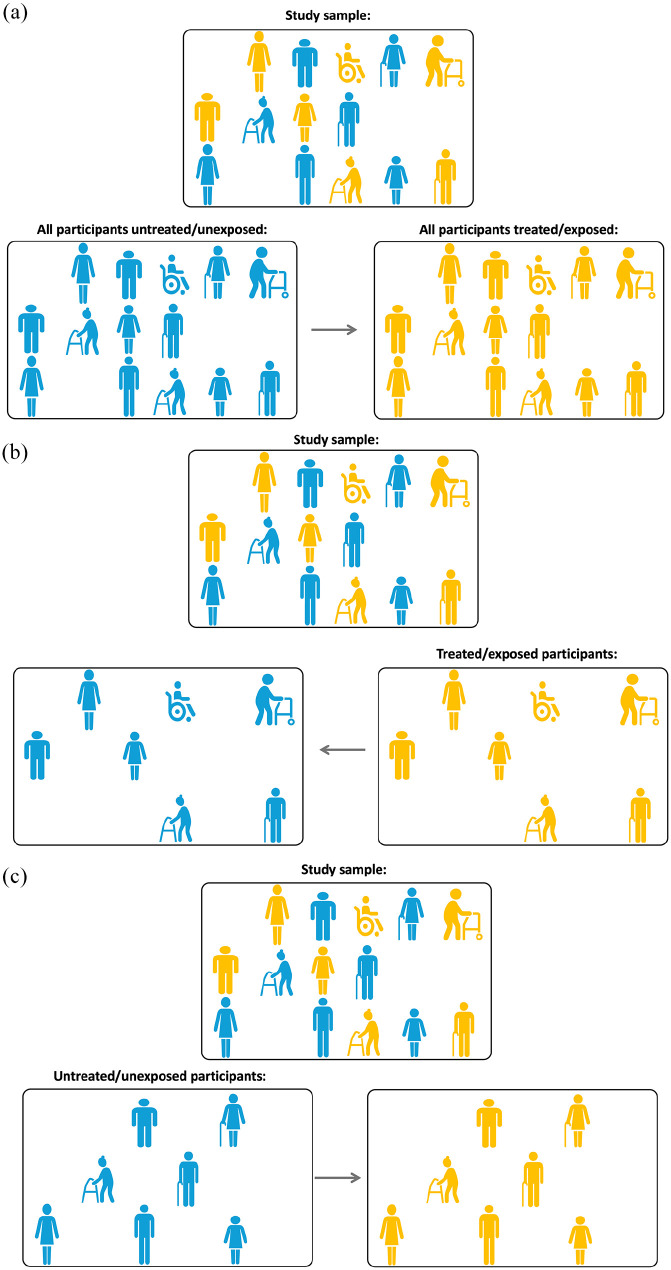
(a) Illustrating Point 4. Average treatment effect, ATE: what if all the participants in the study sample were exposed (treated, yellow) versus unexposed (blue)? (b) illustrating Point 4. Average treatment effect for the exposed (treated), ATT: what if the exposed (treated, yellow) participants in the study sample were actually not exposed (blue)? and (c) illustrating Point 4. Average treatment effect for the unexposed (untreated), ATU: what if the unexposed (blue) participants in the study sample were actually exposed (treated, yellow)?

If we classify the participants who received the MSU care as *treated* (often referred to in the literature as *exposed)* and those who received the standard ambulance care as *untreated* (or *unexposed)*, the answers to Questions 1–3 will be provided by the following effects:

- Question 1: *Average Treatment Effect (ATE).* The ATE is the average effect, in the population, of moving all the participants from being untreated/unexposed to treated/exposed as visually illustrated in Figure 4(a).
- Question 2: *Average Treatment Effect for the treated/exposed (ATT).* The ATT is the average treatment effect in the subpopulation that received the treatment/exposure as visually illustrated in Figure 4(b).
- Question 3: *Average Treatment Effect for the untreated/unexposed (ATU).* The ATU is the average treatment effect in the subpopulation that did not receive the treatment/exposure as visually illustrated in Figure 4(c).

Which treatment effect is of specific interest depends on the clinical context and the nature of the causal inference research question. Consider some illustrative scenarios adapted from Austin^18^:

- Scenario 1: The researchers are interested in the effect of being prescribed antiplatelet therapy, statins and/or antihypertensive therapy at hospital discharge in a sample of patients discharged from hospital with a diagnosis of ischemic stroke. The relevant causal question is about the effect of moving a population of discharged ischemic stroke patients from being untreated to treated and what outcomes this would generate. The ATE is the effect of interest here.
- Scenario 2: The researchers would like to compare the outcomes between various forms of secondary stroke prevention in the absence of atrial fibrillation: for example, aspirin, clopidogrel, or a combination of aspirin with dipyridamole. Both ATE and ATT could be of interest here: ATE describes how outcomes would change if a policy becomes that all patients eligible for either therapy were only offered a specific one of these; while ATT would tell us what was the effect of treatment for those who were given a specific treatment (e.g. clopidogrel).
- Scenario 3: The researchers would like to examine the effect of a structured, intensive cardiovascular rehabilitation program on reducing cardiovascular events - the intervention where, although many subjects are potentially eligible, only a small number of subjects elect to undergo the intervention. Here, ATT may be of greater interest as it estimates the effect of the program on those subjects who elect to participate.

### Recommendation 4.1

The type of causal effect that is of specific interest for a given study heavily depends on the clinical context and the nature of the causal inference research question. It should be clearly presented alongside the causal inference research aim.

## Point 5: Overview of biases in descriptive epidemiology and causal inference studies

Bias in research studies can be broadly defined as systematic errors introduced into components of the research process. Bias favors one outcome or answer over others.^19,20^ These errors can occur during sampling, measurement, testing, or reasoning. Both descriptive epidemiology/associational and causal inference studies that use observational data are subject to various kinds of bias.

Of course, any empirical study with the aim to generalize from a sample is subject to a random error that can affect the precision of the key estimates such as rates, proportions, or association measures. Although this can be a threat to study validity, such random error is not considered a bias as it does not have a systematic nature and it affects precision of the estimate rather than its accuracy.

### Biases relevant for both descriptive epidemiology and causal inference studies

#### Sampling bias

The research aim of a descriptive epidemiology study is to characterize the distributions of health, disease, and harmful or beneficial exposures in a well-defined population *as they exist*, focusing on what is actually observed. Sampling bias is a key concern for such studies. The descriptive research question is typically asked at the level of target (aka source) population, for example, the researchers would like to estimate the incidence of ischemic stroke in hypertensive people above 60 years of age. This may be the population of a particular world region, country, or a state within, or it could be a given community or location. In order to achieve their aim, researchers need to sample appropriately from the identified population to ensure the obtained estimates of risk are representative of the source population. To ensure the data generated from a study sample can support valid reasoning for the purposes of the research question, researchers need to ensure *a well-defined target (source) population* (as defined in RECORD guidelines and discussed earlier in Point 1) and *a valid sampling strategy*.

*Measurement error* and *nonrandomly missing data* on the outcome or key covariates are the kinds of bias that can affect both descriptive epidemiology/associational and causal inference studies. Measurement bias can arise due to an error in assessing an exposure, outcomes or covariates. These errors can be independent of each other, or may depend on exposure or outcome (aka differential errors). For example, in a case-control study of alcohol consumption and stroke, there is a potential for recall bias when reporting of alcohol use according to presence or absence of stroke. As such, an association between the reported measure of alcohol use and stroke may not only be different to a causal effect of true alcohol use, but also presents a biased outcome for the aim of a descriptive epidemiology/associational study of the association between true levels of alcohol use and stroke.

In summary, a valid answer to a descriptive epidemiology/associational research question requires appropriate sampling, valid measurement of the outcome and any covariates, and appropriate data analysis.^5^ Strengthening the Reporting of Observational Studies in Epidemiology (STROBE guidelines)^2,3^ provide a detailed account of potential biases and threats to study validity that may accompany these activities.

### Biases specifically relevant for causal inference studies

#### Confounding bias

Confounding by indication occurs when a specific condition, illness, or patient characteristic influences both the probability of receiving a particular treatment or intervention (exposure) and the outcome of interest. Thus, confounding occurs because of the common (shared) causes of exposure and outcome. For example, a spurious association between carrying a cigarette lighter (exposure) and having an increased risk of stroke event (outcome) may be claimed if the common underlying cause of smoking is not considered and controlled for appropriately. Another example of confounding is when stroke onset-to-treatment time may confound the association between the kind of ischemic stroke reperfusion therapy (exposure) and functional outcome at 90 days post-stroke (outcome) as it may influence both the exposure (the choice between endovascular treatment alone vs in combination with thrombolysis) and the outcome (through expansion of core damage over time). This example is discussed in greater detail in Point 7, where we specifically address the issue of confounding and how to deal with it appropriately in the context of causal inference on observational data, using Directed Acyclic Graphs (DAGs).^21^

#### Selection bias

Arises when a variable that is a common effect of both the exposure and the outcome of interest was controlled for/conditioned on by, for example, stratification/restriction/sample selection, regression adjustment, or matching. Selection bias may generate spurious associations. As an example, consider the situation where stroke survivors with or without a specific prognostic biomarker are undergoing various amounts of post-stroke rehabilitation physiotherapy. We know that being biomarker positive and receiving more therapy are mutually independent, that is, biomarker positive stroke survivors do not get extra therapy, nor vice versa. We also know that being biomarker-positive and receiving more therapy are each associated with better recovery outcome. If we choose only stroke survivors with good recovery (i.e. we condition our sample on the good recovery outcome) and we establish that these participants are biomarker-negative, we will be concluding that they must have received extra therapy to get better recovery. Similarly, if we choose participants with better recovery but know that these participants did not receive additional therapy, we will be concluding that they are biomarker-positive. Thus, being biomarker positive and receiving more therapy no longer appear independent. A spurious association between being biomarker positive and having greater amounts of therapy is established once we condition on the positive recovery outcome. Specific examples of selection bias include non-randomly missing data bias, survivor bias, participation bias, self-selection bias, etc.

Hernan et al^22^ provide a framework to describe and prevent biases that result from a failure to align start of follow-up, specification of eligibility, and treatment assignment, and suggest analytic approaches to avoid these problems in causal studies using observational data via *Target Trial* specification as further discussed in Point 6. A detailed discussion of Quantitative Bias Analysis (a set of methods to quantify the potential impact of residual bias that has not been accounted for through study design or statistical analysis), is beyond the scope of this discussion, but an interested reader is referred to^19,23^ for informative and relevant reviews.

### Recommendation 5.1

Stroke researchers should be mindful that some biases that arise in descriptive epidemiology/associational observational studies are different to, or behave differently from, biases in causal inference studies.

### Recommendation 5.2

While confounding bias is not relevant for descriptive epidemiology/associational observational studies, other kinds of biases, such as selection and measurement bias, should be actively considered and mitigated.

## Point 6: Defining causal effect with the target trial emulation approach

Compared to Rubin’s counterfactual framework discussed in Point 3, that provides a broad structure for understanding causal inferences, a more recent development is the *Target Trial* approach proposed by Hernan et al.^7,24–26^ The *Target Trial* emulation approach provides a specific methodology within Rubin’s framework that enforces a detailed description of the protocol describing a hypothetical randomized trial that the researchers would have wanted to conduct if it were possible, practical, and ethical to conceptualize the exposure of interest as an experimental intervention. For example, consider that we intend to undertake a causal inference study of an effect of alcohol consumption (exposure of interest) on the risk of stroke (outcome of interest). It would be unethical to conduct a real-life clinical trial in which participants are randomized to various levels of alcohol consumption, including potentially unsafe ones, due to existing knowledge about harms of such consumption. Instead, “conducting” a hypothetical randomized clinical trial of this kind via the Target Trial approach promotes explicit articulation of the underlying assumption of the causal effect that researchers aim to estimate.

A recent example of the application of the target trial within stroke is the PRECISE study – designed to evaluate the effect of a government policy to incentivize the use of chronic disease management in primary care of people after stroke, using linked registry and government-held administrative datasets.^27^ Using the framework described by Hernan, the authors described explicitly the seven key components of the study design as outlined in Table 1, allowing real-world examination of the population effect (ATE) of an already implemented strategy. The study cohort was limited to individuals who fulfilled the eligibility criteria for receiving the policy under investigation, specifically those residing in the community and expected to survive the exposure period. The principles of a clinical trial were also emulated by including only individuals who had the potential to be prescribed the policy of interest, that is, those who consulted a primary care physician during the exposure period. To mitigate survivor bias, only individuals who survived the exposure period were included in the analysis of outcomes. The utilization of data on health insurance claims for one or more chronic disease management plans for evaluating the population-level effectiveness of a policy, rather than evaluating the clinical intervention that the policy was designed to support, enabled reliable identification of the exposure. Likewise, the outcome of death was ascertained in a blinded manner using the national death index, ensuring reliable identification of this outcome. As highlighted by Franklin et al,^28^ accurate specification of the comparators and end points are critical to agreement between RCT results and those of the observational simulation.

**Table 1.** Emulated target trial specification.

Protocol component	Emulated trial specifications
Eligibility criteria	Adults admitted to hospital with a clinical diagnosis of stroke or TIA who survived to 18 months post-stroke, had at least one GP claim in the exposure period and were living in the community (i.e. not in residential care) and not admitted to palliative care.
Treatment strategies	*Exposure group:* had a Medicare claim for one or more chronic disease management plans (or review) during the exposure period
	*Comparator group:* Saw the GP at least once but did not receive a chronic disease management plan (or review) during the exposure period
Assignment procedures	A propensity score (*N* = 42 covariates) with inverse probability treatment weights (IPTW) was generated and balance in baseline variables between those with and without a claim was assessed in the weighted sample
Follow-up period	Participants were followed from the start of the 12 month outcome period to the end of the outcome period.
Outcome	Death, blinded using data from the National Death Index
Causal contracts of interest	Intention to treat analysis, per protocol analysis
Analysis	Multi-level mixed effects Cox proportional regression analysis with patient and health service region as random effects. Models were weighted using the IPTW, with year as a covariate. A range of subgroup and sensitivity analyses were performed.

Even in situations where a hypothetical intervention that reflects the exposure of interest is ill defined, the application of the Target Trial approach still minimizes the potential for a range of biases.^26^ Moreover, it can facilitate identification of biases that may not be possible to control within the classic observational framework and could otherwise go unnoticed. In particular, Hernan et al^22^ provide a *Target Trial* based framework to describe and prevent biases for a Target Trial and suggest relevant analytic approaches. It is critical that analyses of observational data undergo appropriate and extensive robustness checks to understand the potential impact of these biases on the effect of interest. This may involve strategies such as performing multiple target trials under different scenarios, investigating differences in the ATE or ATT across subgroups, or conducting a systematic bias analysis. These issues are discussed in greater detail in Points 7, 9, and 10.

### Recommendation 6.1

A *Target Trial* approach should be used to design and conduct causal inference studies on observational data as its use helps avoiding common methodological pitfalls associated with observational studies and increases study validity through identification and minimization of different kinds of biases.

## Point 7: Directed acyclic graphs for articulating and communicating causal hypothesis and minimizing bias

Studies intended to estimate the causal effect of an exposure on an outcome must eliminate multiple biases, such as bias by indication and noncausal sources of association. The more successful the elimination process, the higher the certainty that the established statistical association between an exposure and outcome provides an unbiased estimate of a causal effect.

The remainder of the discussion presented in this point constitutes an adaptation to stroke care context of the tutorial by Digitale et al^21^ that can be consulted for further detail and relevant references.

*Directed Acyclic Graphs* (DAGs) provide an external and explicit graphical representation of the causal hypotheses about the clinical or care system of interest. DAGs consist of variables (also referred to as nodes; e.g. representing treatments, exposures, health outcomes, or patient characteristics) and arrows (aka edges), which depict known or suspected causal relationships between variables.

Consider the following hypothetical causal inference on observational registry data study where patients receive ischemic stroke reperfusion therapy (e.g. thrombolysis vs endovascular thrombectomy vs both treatments) to improve their functional outcome 90 days post-stroke.

- Due to the observational nature of the study, treatment assignment is not random. It is influenced by historical limitations on stroke onset-to-treatment times. For example, more participants undergoing thrombolysis presented earlier post-stroke and more participants treated with thrombectomy alone presented later.
- Stroke onset-to-treatment time is also known to influence the infarct core size (with the core size increasing over time). That, in turn, is hypothesized to influence the functional outcome 90 days post-stroke. Outcome is also influenced by the patients’ age.
- In addition to directly influencing functional outcome 90 days post-stroke, ischemic stroke reperfusion therapy is hypothesized to influence it via the reperfusion success, for example, measured as a TICI score post-procedure.
- Ischemic stroke reperfusion therapy may also affect the patient’s acute length of stay (Acute LOS), perhaps also influenced by whether or not the patient is receiving stroke unit care.
- It is also hypothesized that stroke unit care directly causally affects functional outcome 90 days post-stroke.
- Finally, it is hypothesized that functional outcome 90 days post-stroke has a direct causal link to patients’ quality of life measured at 12 months post-stroke.

The hypothetical study aim is to estimate the causal effect of ischemic stroke reperfusion therapy (e.g. thrombolysis vs endovascular thrombectomy vs both treatments) on functional outcome 90 days post-stroke.

**Figure 5.**
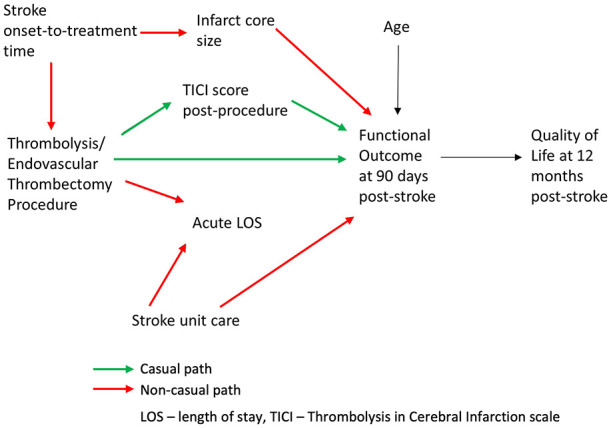
Illustrating Point 7. Directed Acyclic Graph (DAG) representing causal hypothesis for the effect of a stroke reperfusion therapy on the functional outcome at 90 days post stroke. Causal paths from the exposure (Thrombolysis/Endovascular Thrombectomy Procedure) to the outcome (Functional Outcome at 90 days post-stroke) are shown in green, non-causal paths are shown in red.

The above situation is represented as a DAG in Figure 5 that explicitly articulates the formulated causal hypothesis for the effect of a stroke reperfusion therapy on the functional outcome at 90 days post stroke. To use the DAG as a tool for causal reasoning, the following key terms and concepts are required:

- *Paths* are sequences of arrows, in any direction, that connect two variables
- *Paths* can be *causal* or *non-causal*: *causal paths* are formed by the arrows pointing in the same direction, that is, when each preceding variable is causing the subsequent variable; *non-causal paths* are formed by the arrows going in opposing directions, that is, they are containing confounders and/or colliders
- To identify a *causal path* from *exposure* (e.g. *Thrombolysis/Endovascular Thrombectomy Procedure*) to *outcome* (e.g. *Functional Outcome at 90 days post-stroke*), all the *non-causal paths* between the former and the latter should be *blocked* and all of the *causal paths* should be *open* (non-blocked).
- *Confounding* occurs because of the common (shared) causes of *Thrombolysis/Endovascular Thrombectomy Procedure* (exposure) and *Functional Outcome at 90 days post-stroke* (outcome). *Stroke onset-to-treatment time* confounds the association between *Thrombolysis/Endovascular Thrombectomy Procedure* (exposure) and *Functional Outcome at 90 days post-stroke* (outcome).
- *Mediators* are caused by the exposure and, in turn, are causing the outcome. For example, *TICI Score post-procedure* partially mediates the effect of *Thrombolysis/Endovascular Thrombectomy Procedure* on *Functional Outcome at 90 days post-stroke.*
- *Colliders* (variables having two arrows pointing to them) on a path *block* that path. A presents a common effect of two variables, one of which is either the exposure of interest or a cause of the exposure, and the other is either the outcome or a cause of the outcome. For example, *Acute LOS* is a *collider* on a non-causal path from *Thrombolysis/Endovascular Thrombectomy Procedure* to *Stroke Unit Care* and, therefore, is also a *collider* on a non-causal path from *Thrombolysis/Endovascular Thrombectomy Procedure* to *Functional Outcome at 90 days post-stroke.* Thus, *Acute LOS* *blocks* the non-causal path from the exposure to the outcome unless it (or its consequence) is controlled for.
- An *Instrumental variable* is a variable that is a cause for the exposure but does not have any relationship with outcome other than that via exposure. For example, were the study under consideration to be a randomized one, the potential *Randomization* variable would have been considered instrumental for the effect of *Thrombolysis/Endovascular Thrombectomy Procedure* on *Functional Outcome at 90 days post-stroke.*
- The variable *Quality of Life at 12 months post-stroke* is a *descendant* of *Functional Outcome at 90 days post-stroke.*
- *Effect modification* happens when the effect of exposure on outcome varies across strata of a third variable .^29^ For example, the effect of Thrombolysis/Endovascular Thrombectomy Procedure on *Functional Outcome at 90 days post-stroke* may vary depending on the value of *Infarct Core Size*, which means that *Infarct Core Size* may be an effect modifier. *Effect modifiers* are variables that also cause the effect and, therefore, modify the relative or absolute effects of other causes on at least one effect measure scale (additive or multiplicative).

Any statistical association found between the exposure and the outcome can be considered an unbiased estimate of a causal effect of the exposure on the outcome if both of the following conditions are true: (1) all noncausal paths from the exposure to the outcome are blocked and (2) no causal paths from the exposure to the outcome are blocked.^21^ Therefore, to identify causal effect of *Thrombolysis/Endovascular Thrombectomy Procedure* (exposure) on *Functional Outcome at 90 days post-stroke* (outcome), *all the non-causal paths* between the exposure and the outcome *should be blocked* and *all of the causal paths should be open* (non-blocked). Opening or blocking relevant causal paths can be achieved via controlling for/conditioning on an appropriately chosen variable(s) which happens when we use any technique or method that fixes its value, for example, by stratification, restriction, sample selection, regression adjustment, or matching.

The following paths leading from the exposure to the outcome can be identified in Figure 5:

### Causal paths

- *Thrombolysis/Endovascular Thrombectomy Procedure* → *TICI Score post-procedure* → *Functional Outcome at 90 days post-stroke*
- *Thrombolysis/Endovascular Thrombectomy Procedure* → *Functional Outcome at 90 days post-stroke*

### Non-causal paths

- *Thrombolysis/Endovascular Thrombectomy Procedure ← Stroke onset-to-treatment time → Infarct Core Size → Functional Outcome at 90 days post-stroke* (can be blocked by conditioning on/controlling for *Stroke onset-to-treatment time* or *Infarct Core Size*)
- *Thrombolysis/Endovascular Thrombectomy Procedure → Acute LOS ← Stroke Unit Care → Functional Outcome at 90 days post-stroke* (is blocked unless we condition on/control for *Acute LOS*)

Thus, given the causal hypothesis presented in the DAG in Figure 5, in order to estimate the causal effect of *Thrombolysis/Endovascular Thrombectomy Procedure* on *Functional Outcome at 90 days post-stroke*, the following actions to control for/condition on some variables will be needed:

- It is *necessary to control for common causes or other variables on the non-causal path*. Controlling for either *Stroke onset-to-treatment time* or *Infarct Core Size* should be adequate to eliminate confounding due to *Stroke onset-to-treatment time* by blocking the confounding path between the exposure and the outcome. For example, in situations where it is easier to obtain accurate measurements of *Infarct Core Size* than those for *Stroke onset-to-treatment time* (e.g. in wake-up stroke patients), controlling for *Infarct Core Size* could be preferred.
- *Mediators should not be controlled for* to estimate the total causal effect of the exposure on the outcome. For example, controlling for *TICI Score post-procedure* will produce a biased estimate of the effect of exposure on the outcome.
- *Colliders (or their consequences) should not be restricted on or controlled for*, as such controlling or restricting will unblock the non-causal path and create a bias in the form of spurious association between the exposure and the outcome. For example, controlling for *Acute LOS* will introduce a bias to the estimate of the effect of exposure on the outcome.
- *Descendants* (e.g. *Quality of Life at 12 months post-stroke*) *should not be adjusted for, stratified on, or in any way conditioned on.*

DAGitty (https://www.dagitty.net/) is a free browser-based environment for creating, editing, and analyzing directed acyclic graphs for minimizing bias in empirical studies in epidemiology and other disciplines.^30^ The related webpage also provides a list of valuable resources for further leaning about DAGs and their role in articulating and communicating causal hypotheses and preventing confounding.

### Recommendation 7.1

DAGs should be adopted to clearly articulate and communicate causal hypotheses for causal inference studies on observational data as a DAG can identify variables that: (a) should be controlled for in the design or analysis phase to eliminate confounding and some forms of selection bias and (b) should not be controlled for to avoid biasing the analysis.

### Recommendation 7.2

As causal hypotheses expressed as DAGs reflect the state of prior knowledge about the system under study, in situations where such knowledge is limited and several alternative DAGs are plausible, the differences in the outcomes that result from alternative analyses should be systematically investigated and related uncertainties should be clearly communicated.

## Point 8: Stratification and adjustment in observational epidemiology studies: what it means and whether it is needed

Quite often when reading the review reports on their manuscript, stroke researchers find a suggestion by the reviewer to consider a range of relevant factors originally unaccounted for in the analysis and to run a respective multivariable regression model that would adjust the estimate of interest for these factors.

As discussed earlier in Point 2, Garcia-Esperon et al^4^ used INSPIRE registry data to evaluate the association of endovascular thrombectomy (EVT) with functional outcome in patients with a baseline ischemic core volume > 70 ml. As a part of the peer-review process, they received the following recommendation from an anonymous reviewer (clarifying words added in <. . .>):

“*As pointed out by the Authors, compared with non-EVT patients, EVT patients were significantly younger, had a lower time from* <*stroke*> *onset to CT, a lower premorbid mRS, a significantly higher median baseline ASPECTS score, a smaller median core volume with a larger penumbra area, and had a lower proportion of internal carotid artery* <*cases*>. *It is acknowledged that since in this study the comparison is between patients treated* versus *those non treated with EVT, there could be differences in terms of a lower time from stroke onset to CT in EVT patients and of lower baseline ASPECTS score, smaller core, and larger penumbra. However, it could be not determined with certainty that these differences were due to the fact that CT were performed earlier than in non-EVT patients. These baseline radiological characteristics are important confounding factors that could influence clinical outcome and that, except for age, were not taken into account in the multivariate (sic) analysis. In order to address this issue, the Authors should perform, as sensitivity analysis, a further multivariable analysis including at least baseline core volume and the presence of carotid artery occlusion*.”

In order to provide further context, three important points need to be clarified:

- According to the definition of the descriptive epidemiology/association research aim provided earlier in Point 2, the researchers were interested to compare the distribution of functional outcomes between EVT and non-EVT in patients with a baseline ischemic core volume > 70 ml under exposure conditions that these patients had *actually* received (having undergone EVT) to those who actually did not;
- As discussed in Points 3, in causal inference studies, confounding occurs in situations where the effects of the exposure on a given outcome are mixed in with the effects of an additional factor (or set of factors) that distort the true relationship (i.e. the association is spurious).^31^
- The purpose of including all important factors in a multivariable (multiple) regression model in addition to the exposure variable (EVT) is to obtain *adjusted* estimates. The broad meaning of adjustment (aka “controlling for/conditioning on other variables” or “stratification” in case of discrete factors) in this context is to obtain the estimate of interest holding all other independent variables constant. Such estimates are often interpreted as “assuming no changes in all other inputs.” Although in our example case above, adjustment is achieved using a regression model, any method that “fixes” the value of covariates, for example, stratification, sample selection, or matching, may be used for this purpose.

Putting the above points together, the anonymous reviewer’s suggestion was for the study that had an expressed research descriptive epidemiology/association aim, instead to modify the association of interest being estimated by the authors and estimate the association adjusted for several important confounders. Given the clearly formulated descriptive epidemiology aim of the study (to estimate associations), was this a correct suggestion and should the suggested analyses have been performed? After all, every factor mentioned in the recommendation seems relevant and logically sound: they each may have a potentially modifying effect on the endpoint being estimated.

The answer is that following the reviewer’s recommendation in this context would not have been right. There are at least three reasons for this:

- The first reason is provided in Point 2: there are fundamental differences between descriptive epidemiology/association and causal inference questions. Confounding is a causal concept that does not apply to associations.^7^ According to Hernan (emphasis added): “There is no such thing as a “spurious association” unless we use the term to mean an *association* that cannot be *causally interpreted*—but then the goal of the analysis would be causal, not associational.” As the pre-specified aim of the observational analysis was purely associational, no adjustment for confounding is necessary. In our example, since the authors aimed to quantify the association between EVT and functional outcomes, while the clinical interpretation of such an association may be limited, this is simply estimated from the data.
- The second reason is that any statistical relationship is expected to be influenced by other variables. Contrary to the widely held belief that statistical adjustments can only assist in avoiding spurious associations, as discussed in Point 7, the reality is that the use of such adjustment without appropriate methodological foundation, actually increases the risk of introducing meaningless associations, for example by accidentally opening a collider path and creating an association that is spurious.
- In some cases (e.g. as discussed by Fox et al^5^), adjusting for an important “marker” such as a geographic area, may “adjust away” the underlying socioeconomic reasons that drove people to live where they do (such as income) and that influenced other health conditions in residents of those places (such as availability of healthy foods or health services).^5^ Therefore, for a descriptive epidemiology study, inappropriate adjustment can make it harder to see the magnitude of disparities.

Does it mean then that there is no space for stratified or adjusted analyses in descriptive epidemiology/associational studies? There are important reasons descriptive statistics are sometimes more useful after adjustment for or stratification by some variables that are known to be prognostic for the outcome and whose distribution differs across populations.^32^ These reasons include the research aim to assess heterogeneity across and within patient groups or to identify groups at high risk of disease.^5^ For example, Garcia-Esperon and colleagues^4^ estimated the association between EVT and the outcomes, adjusted for the following pre-specified covariates known to be prognostic for mRS at 3 months: age, pre-morbid modified Rankin Scale (mRS), and National Institutes of Health Stroke Scale score (NIHSS).

It is important to reiterate that stratifying/adjusting in an ad hoc manner increases the risk of claiming meaningless associations. The choices about variables to consider for stratification and/or adjustment strata should be driven by theory and context. This consideration is even more important for causal inference studies as discussed in Points 7 and 9. A clear analytical plan for a descriptive epidemiology/association study should be developed. Lack of such a plan can lead to poor execution and a confused interpretation that is neither descriptive nor causal in its nature, for example studies framed as “risk-factor” analysis.^5,33,34^ One such example is the study by Fekadu and colleagues who studied risk factors, clinical presentations and predictors of stroke among adult patients admitted to stroke unit of Jimma university medical center, south west Ethiopia. Due to combining a number of different research aims in the same study, the authors used multivariable logistic regression analysis model with backward stepwise selection to identify risk factors. While the proposed analytical approach may be appropriate in some cases for achieving the research aim of selection of stroke predictors, such an approach cannot produce meaningful outcomes for either an associational or causal inference research question.

In summary, in descriptive epidemiology/associational studies, stratification or adjustment does not equate to “confounding control,” simply because confounding is not relevant in descriptive epidemiology/associational studies.^5^ The notion of a “spurious association” is meaningless as any association is affected by other variables and does not become “spurious” until given a causal inference meaning. The question of interest should guide the decision as to whether it is meaningful to adjust or stratify the analysis. Using adjusted analyses to counter a possibility of confounding when the research aim is to investigate association should not be promoted.

### Recommendation 8.1

In observational epidemiology/associational studies multivariable analysis with the aim to adjust for confounding should not be used.

### Recommendation 8.2

The research aim and research question should guide the decision of whether to stratify/adjust in the descriptive epidemiology question. No adjustment should be performed without a clear rationale and a detailed explanation of the meaning of the results “with adjustment.”^5^

## Point 9: Selecting a confounder adjustment set for causal studies and positivity assumption

In contrast to association studies, if the research aim of the observational analysis is causal, adjustment for confounding is generally necessary. As discussed in Point 7, DAGs assist with identification of which variables should or should not be controlled for in the design or analysis phase, to eliminate both confounding and some forms of selection bias and to avoid biasing the estimated causal effect. In particular, once identified using an appropriately-designed DAG, it is necessary to control for common causes or other variables on the non-causal path from the exposure to the outcome. Mediators, colliders, and descendants should not be adjusted/controlled for, stratified on, or in any way conditioned on. Therefore, when using DAG graphical rules, the quality of decisions regarding which adjustment covariates to select is determined by the validity of the underlying causal hypothesis.

While encouraging stroke researchers to put maximum effort into formulating relevant DAGs to reflect the causal hypotheses, it is important to recognize that complete causal knowledge is often unavailable. A review of various developments in causal inference on the topic of confounder selection is presented by VanderWeele and VanderWeele.^35^ Some of the practices of covariate selection for confounding control that are *not recommended* (as they can result in a biased estimate of the causal effect) include:

- Controlling for any variable that is prior to the treatment or exposure under study – as this may include controlling for a collider, thus opening a non-causal path and introducing bias as discussed in Point 7;
- Adjusting for *all* pre-exposure covariates that are presumed to be common causes of exposure and outcome. In some cases the researchers may not have the data for some of the covariates presumed to be common causes but have instead a different set of covariates that could be considered as proxies and could be sufficient to control for confounding.;
- Data-driven approaches (such as forward/backward selection procedures or when a covariate is selected if its inclusion changes the estimate of the causal effect for the exposure by more than some threshold) – as such procedures do not provide a foundation for causal reasoning. They only help with choosing the most parsimonious models once the process of casual reasoning for covariate selection has been applied.

In situations where it can be assumed that “knowledge is available for each covariate whether it is a cause of the exposure, and whether it is a cause of the outcome.,” VanderWeele and VanderWeele^35^ presents an attempt to devise a pragmatic strategy for covariate selection. The *recommended practice* is:

- to control for each covariate that is a cause of the exposure, or of the outcome, or of both;
- to exclude from this set any variable known to be an instrumental variable (as defined in Point 7); and
- to include as a covariate any proxy for an unmeasured variable that is a common cause of both the exposure and the outcome.

Applying these rules to the case presented in Point 7 depicted in Figure 5, the following variables could be included in a causal effect estimation model as covariates: *stroke onset-to-treatment time* (or *infarct core size* as a proxy for time should the time data be unavailable), *age*, and stroke unit care.

The above considerations for selection of adjustment covariate selection were aimed at addressing the *no unmeasured confounding* and *correct causal model specification* assumptions for causal reasoning discussed in Point 3. An equally important assumption underlying the causal reasoning process that also has direct implications for the covariate selection procedure is that of *positivity*.^16^

**Figure 6.**
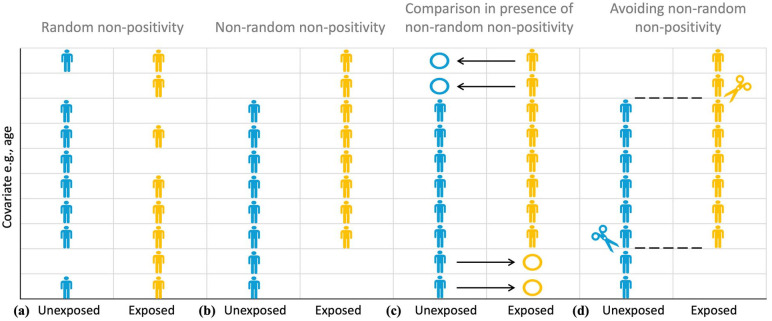
Illustrating Point 9. The difference between random (a) and non-random (b) non-positivity. Positivity assumption requires that there be both exposed (yellow) and unexposed (blue) participants at every combination of the values of the observed confounders (e.g., age) in the study sample. Non-random (deterministic) non-positivity is illustrated here by older study participants only belonging to the exposed group, making it impossible to elicit a meaningful estimate of exposure effect for such participants due to the absence of a meaningful comparator in the unexposed group (c). The study sample can be trimmed or matched to avoid regions of non-overlap that emerge as the result of non-positivity (d).

*Positivity*, or *the experimental treatment assignment assumption*, requires that there be both exposed and unexposed participants at every combination of the values of the observed confounders in the study sample. Figure 6 illustrates various aspects of positivity assumption using age as an example of a confounding covariate. A randomized clinical trial satisfies the *positivity* assumption as at randomization, every participant has a known probability of each exposure/treatment under study, but this is not necessarily the case in the observational studies.

Note that the positivity assumption does not require there to be equal (or indeed even similar) numbers of exposed and unexposed participants at every combination of the values of the observed confounders in the study sample. What is required is that, based on the causal hypothesis or the observed data, for both the unexposed and exposed groups, the probability of a participant with a given combination of the values of the covariates of interest belonging to a group should be non-zero, that is, positive, hence the *positivity* name. A probability of belonging to a particular group as the function of the values of the covariates of interest is quantified using a *propensity score* and is discussed in detail in Point 10.

It is possible to distinguish between *random* and *non-random* (*deterministic)* violations of the positivity assumption. This distinction is illustrated in Figure 6. *Deterministic non-positivity* (Figure 6(b)) is present when study participants with at least one combination of confounders (e.g. age) *cannot* belong to at least one of the groups characterized by levels of exposure. For example, considering the case presented in Point 7, based on the current guidelines, participants presenting over 24 h since stroke event, as a rule, will not be eligible for thrombolytic therapy, while participants without a large vessel occlusion, as a rule, will not be eligible for thrombectomy. Therefore, in the case of the INSPIRE registry discussed earlier in Points 2 and 8, were a causal inference question about the effect of a reperfusion therapy on the functional outcome to be asked, particular care would have to be exercised to ensure the absence of *deterministic non-positivity* in the stroke-to-treatment time for the treatment exposure.

In contrast, random *non-positivity* emerges when, although potentially possible, no participants with at least one combination of confounders happen to be observed in one or more groups characterized by levels of exposure (Figure 6(a)). According to Westreich and Cole,^16^ random non-positivity can be classified further by whether or not the combinations of confounders that create regions of non-positivity are surrounded by the regions of positivity (*internal* vs *external*). For example, it may happen that no participant aged between 70 and 73 years is observed in the endovascular thrombectomy group, but there are participants who are either younger than 70 years or older than 73 years. In this case, the region of observed non-positivity based on age is surrounded by respective regions of positivity.

The presence of deterministic non-positivity is of serious concern, as ignoring it can lead to uninterpretable causal effect estimates (Figure 6(c)).^16^ Consider, for example, the interpretation of the reperfusion treatment therapy effect adjusted for stroke-to-treatment time that systematically violates the non-positivity assumption because no participant is exposed to thrombolysis therapy beyond 24 h after stroke onset. As discussed in Point 8, such an adjusted effect should be interpreted as the effect of endovascular thrombectomy compared to that of thrombolysis “assuming similar time-to-treatment,” while these times are clearly dissimilar to the point of “extrapolating into emptiness”: there is no participant with time-to-treatment above 24 h in the thrombolysis group that can be compared to otherwise similar participants undergoing endovascular thrombectomy. Such an effect estimate will clearly not be valid. *Random non-positivity* is harder to assess and inevitably involves qualitative judgment and expert opinion.

A recommended way to mitigate the violations of positivity is based upon careful statement of the causal inference research question, particularly in relation to the target population for causal inference.^16^ The study sample can be trimmed or matched to avoid regions of propensity score non-overlap that emerge as the result of non-positivity (Figure 6(d)). While such reductive changes in the study sample have obvious implications for restricting the target populations of interest, as discussed in Point 1, not using all the routinely collected data is not only non-problematic, but is natural and perfectly acceptable. The purpose of data collection is different to that of a causal inference study and the database population is naturally different to the study population.

In cases where non-positivity is assessed to be both random and internal (Figure 6(a), e.g. positivity at ages 56–65 and 71–80 years but not at ages 66–70 years), Westreich and Cole^16^ recommend cautious interpolation or smoothing over the region of non-positivity. If, on the other hand, the non-positivity appears random and external (e.g. non-positivity under 56 years), extrapolation could be risky. This is conceptually more similar to the case of *non-random non-positivity* (Figure 6(b)). Restricting or trimming the sample to include only the participants above 56 years old could be a better approach (Figure 6(d)).

Finally, it is important to emphasize that the selection of a confounder adjustment set for a causal inference model requires trade-offs. Such trade-offs may arise between introducing potential bias from violations of the positivity assumptions (when including a potentially causally important covariate may lead to non-random non-positivity for that covariate) and from confounding due to ignoring important covariates (when excluding a potentially causally important covariate due to non-positivity may lead to inability to appropriately account for confounding), as well as due to the statistical considerations surrounding multivariable regression, in particular, the issues of excessive collinearity. It is an iterative process where alternative causal models should be systematically evaluated and appropriate measures to avoid non-positivity should be implemented.

### Recommendation 9.1

For causal inference studies on observational data, adjustment for confounding is generally necessary. Ideally, it should be based on the causal hypothesis depicted as a DAG using relevant rules. If sufficient causal knowledge is unavailable, a recommended procedure is to control for each covariate that is a cause of the exposure, or of the outcome, or of both; to exclude from this set any variable known to be an instrumental variable; and to include as a covariate any proxy for an unmeasured variable that is a common cause of both the exposure and the outcome.^35^ Data-driven approaches alone should not be used to select covariates to adjust for confounding.

### Recommendation 9.2

Positivity is one of the key underlying assumptions of causal inference. Appropriate methods for managing non-positivity should be used to mitigate relevant biases. These may include trimming, matching, or interpolation.

## Point 10: Analytical methods to reduce bias in causal inference studies

As summarized in Table 2, analytical methods for addressing causal inference questions can be broadly classified into those relying on *fitting the model for the outcome* (such as regression, matching, stratification, G-Computation) and those *modeling the exposure*, that is, estimating the probability (propensity) of a participant being exposed for each individual based on their values of the covariates of interest. The *propensity score* is the probability of being exposed given the values of the covariates of interest. Propensity scores therefore can be used for balancing. Such balancing on the propensity scores is equivalent to balancing on all of the confounders used to estimate the propensity scores, thus reducing the balancing problem to a single dimension. Among the participants with the same propensity score, the distributions of the confounders are balanced between exposure groups, hence their actual exposure is essentially random under the assumption of no unmeasured confounding.

**Table 2.** Analytical methods for addressing causal inference questions on observational data.

Kinds of adjustment	Modeling exposure: propensity scores	Modeling outcomes
Adjustment by conditioning	• *Direct matching* on propensity score	• *Direct matching* on covariates
	• *Stratification* on specific percentiles of the propensity score (e.g., deciles)	• *Stratification* on specific levels of covariates
	• *Regression models* that include a propensity score as covariate either instead of, or in addition to the selected confounders	• *Regression models* that include selected confounders as covariates
Adjustment by standardization in reference to the total-sample confounder distribution	• *Inverse Probability Treatment Weighting (IPTW) based on propensity scores:* achieving total-sample confounder distribution by using the propensity score to reweight exposed and unexposed participants to emulate total-sample confounder distribution	• *G-Computation:* achieving total-sample confounder distribution, as outcome summary measures are taken over all participants in the sample

In addition to the *modeling the outcome* versus *modeling the exposure* classification, analytical methods can be classified into those providing *adjustment by conditioning* versus *adjustment by standardization* as discussed below.

### Adjustment by conditioning: Regression, matching, stratification

Regression provides a model-based way of conditioning on/controlling for/fixing the value of the confounder. For a model with a single covariate, the regression coefficient for the exposure/treatment term estimates the effect of exposure at every value of a covariate. Therefore, the underlying assumption is that the causal effect is the same within all confounder substrata as defined by the covariate. Regression can be used to adjust for any number of confounders, subject to the usual technical constraints on the sample size, model fit and collinearity, the discussion of which is beyond the scope of this primer. The relevant assumption then is that the causal effect is the same within all combinations of the values of the observed confounders in the study sample.

A very important point is that for estimating the causal effect of the exposure on the outcome, *only the regression coefficient for the exposure/treatment term* is of interest, while all other coefficients are just parameters that describe the model’s assumptions. In particular, a coefficient for an adjustment covariate allows the outcome estimate to vary across levels of that covariate while the exposure effect remains the same. These coefficients for adjustment covariates have no substantive meaning for estimating causal effect and are not worth reporting. Moreover, naïve interpretation of “mutually adjusted” measures of causal effect (typically expressed as adjusted mean differences or adjusted odds/risk/hazard ratios) generated through multivariable regression modeling is known to result in substantively wrong conclusions, leading to the phenomenon described in the literature as a “Table 2 fallacy.”^33^

An example may illustrate this better. Consider a potential regression model to address the causal question of the effect of the reperfusion therapy on the functional outcome presented in Point 7 depicted in a DAG in Figure 5. We could include the following variables in a causal effect estimation model as covariates: *stroke onset-to-treatment time, age, and stroke unit care*. The resulting regression model would report the “mutually adjusted” estimated coefficients (e.g. in the form of adjusted odds ratios for achieving *independent function* (mRS0-2)) for *reperfusion therapy*, *stroke onset-to-treatment time*, *age*, and *stroke unit care*. However, as discussed by Westreich and Greenland,^33^ even if the specified model is correct, these coefficients represent different types of causal effects:

- The odds ratio for *reperfusion therapy* can be interpreted as the conditional total effect of *reperfusion therapy* on achieving *independent function.* This means it shows the effect of endovascular thrombectomy compared to thrombolysis at any given level of *stroke onset-to-treatment time, age*, and *stroke unit care*.
- At the same time, the respective adjusted odds ratio for *stroke onset-to-treatment time* cannot have a similar interpretation, that is, *it* *cannot* *be interpreted as a* *total effect* *of onset-to-treatment time* on *achieving independent function*. In the adjusted model, the adjusted odds ratio for *onset-to-treatment time* presents a *direct effect* of time relative to reperfusion therapy. It is the component of the effect of time on achieving independent function that is not mediated via reperfusion therapy. It can be described as the *controlled direct effect of time*, that is, the causal effect of onset-to-treatment time on achieving independent function when reperfusion therapy is held fixed at a given level (either thrombolysis or endovascular thrombectomy), thus blocking the time effect on reperfusion therapy.

Hence, to summarize, reporting “mutually adjusted” coefficients for all covariates included in the causal analysis regression model should be discouraged.

The adjustment for confounding can also be achieved by *conditioning on propensity score* either via *direct matching* on propensity score, via *stratification* on specific percentiles of the propensity score (e.g. deciles), or via *regression model* that would include a propensity score as covariate either instead of, or in addition to the selected confounders.

### Adjustment by standardization

**Figure 7.**
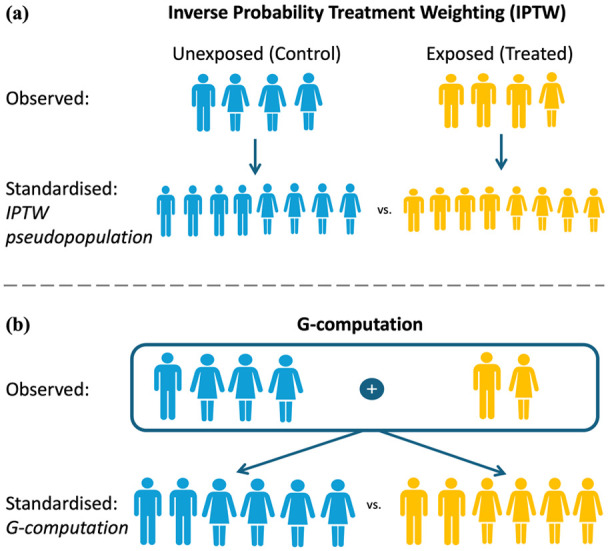
Illustrating Point 10. Standardisation via Inverse Probability Treatment Weighting (IPTW) based on propensity scores (a) and via G-Computation (b). Under IPTW, re-weighting of the observations is aimed to achieve the exposed and unexposed groups being balanced as the re-weighted confounder distribution under both exposed and unexposed scenarios is the same as total sample. Under G-Computation approach, total-sample confounder distribution is aimed to be achieved because outcome summary measures are taken over all participants in the sample.

As discussed in Points 3 and 4, the answer to a causal question requires the researcher to observe all the study participants hypothetically under two or more mutually exclusive exposure conditions in order to derive the relevant measure of effect, such as a difference in mean values or an odds ratio of the outcome between *all the participants* receiving different levels of treatment exposure. In reality, though, groups of participants underwent different exposures. Under the causal inference assumption of *no unmeasured confounding* (discussed in Point 3), *Standardization* refers to the use of statistical modeling to recover the remaining “unobserved” outcomes in reference to the total-sample confounder distribution (Figure 7). Standardization can be achieved by using Inverse Probability Treatment Weighting (IPTW) based on propensity scores (Figure 7(a)) or via G-Computation (Figure 7(b)). As detailed below, under G-Computation approach, total sample confounder distribution is aimed to be achieved because outcome summary measures are taken over all participants in the sample. Under IPTW, re-weighting of the observations is aimed to achieve the exposed and unexposed groups being balanced as the re-weighted confounder distribution under both exposed and unexposed scenarios is the same as total sample.

*G-Computation* estimates summary measures for the outcome at each level of exposure, standardized to the total-sample confounder distribution.^36^ To achieve this, the outcome model built using the principles discussed in Points 7 and 9 is used to estimate outcomes for all participants, regardless of their observed level of exposure, as if they received a particular level exposure. For example, in the case of INSPIRE registry discussed earlier in Points 2 and 8, were a causal inference question about the effect of a reperfusion therapy on the functional outcome to be asked, the probability of achieving a predefined level of function (say, mRS0-2) would have to be estimated for *all the selected participants* irrespective of the type of treatment obtained, first under the assumption that they were treated with thrombolysis and then under the assumption that they were treated with endovascular thrombectomy, and then the average measure of risk difference for all the participants would have to be generated as the measure of the causal effect. Under this approach, total-sample confounder distribution would have been achieved because outcome summary measures were taken over *all participants in the sample*. As part of G-Computation, often the assumptions of causal effect being the same within all combinations of the values of the observed confounders in the study sample is relaxed and relevant interaction terms are included in the statistical model. More information on the practical use of G-Computation can be found in.^37^

Another way to use *adjustment by standardization* is to employ *Inverse Probability Treatment Weighting (IPTW)* based on *propensity scores*. Using this approach, first the inverse of (i.e. one divided by the value of) the probability that each participant received their actual exposure is estimated to understand, based on the values of the selected covariates, how likely it was that a participant should have received the level of exposure that they actually received. Then, a regression model for the outcome is fitted, weighting each observation by the IPTW. The standardization is achieved by using the propensity score to reweight exposed participants to emulate total-sample confounder distribution, then taking the summary measure of interest under that scenario, followed by using (1 – propensity score) to reweight unexposed participants to emulate total-sample confounder distribution, then taking the summary measure of interest under that scenario. Such re-weighting of the observations is aimed to achieve the exposed and unexposed groups being balanced as the re-weighted confounder distribution under both exposed and unexposed scenarios is the same as total sample, consistent with the overall idea of standardization discussed earlier. The described method requires slight modification in terms of how the weights are calculated if instead of the ATE causal effect being of interest, the researchers would like to estimate ATT or ATU effects (see discussion in Point 4). The technical issues such as the use of stabilized or overlap weighting and the need for robust statistical estimators for standard errors when using these procedures are beyond the scope of this primer.^38^ More detailed information on the use of propensity scores for estimating causal effects in the context of neurology can be found in the discussion by Austin et al.^39^

For example, the PRECISE study, introduced in Point 6, used IPTWs for achieving an unbiased estimation of the ATE. In observational studies IPTWs are used to create a pseudo-population where the distribution of observed covariates is balanced between treatment groups, mimicking the conditions of a randomized controlled trial. To ensure minimization of bias, covariates used in IPTW calculations should align with baseline characteristics typically collected in an RCT within a similar population, while also considering additional factors known to influence treatment allocation or the outcome of interest. This can be challenging in observational studies, particularly when utilizing secondary data sources which may not contain the required information. In the PRECISE study, balance across 42 covariates, meeting the criteria above, was achieved by linking data from several different sources. As discussed in greater detail earlier in Point 9, reliability of IPTWs relies on the assumption of *positivity*, meaning that each individual has a non-zero probability of receiving both the treatment and control. Violating this assumption can lead to unstable or unreliable estimates. The application of eligibility criteria imposed by the target trial framework discussed in Point 6 increases the likelihood of fulfilling this assumption.

Where feasible, it is recommended to conduct an analysis of causal contrasts in addition to the primary analysis. This involves simulating a per-protocol analysis, an assessment of effectiveness based on the actual treatment received rather than the group to which participants were randomized. This is particularly challenging in observational studies due to limited information on treatment adherence. To address this limitation, sensitivity analyses can be performed by focusing on a subset of individuals who are deemed more likely to adhere to the treatment protocol based on specific criteria. In the PRECISE study, this involved investigating factors known to be crucial for the optimal provision of the care type associated with the policy of interest, such as the regularity and continuity of primary care physician contacts during the exposure period or regular claims associated with the policy of interest.

In summary, all the discussed analytical methods rely on the same causal assumptions discussed in Point 3. What distinguishes them is that these methods rely on different statistical parametric assumptions, requiring correct specification of the model of interest, that is, whether the exposure or the outcome is being modeled. Standardization propensity score-based methods (such as IPTW) explicitly consider the balance of confounders, essentially creating an analog of a randomized study where no outcomes are considered until the very last step. This effectively separates the design and analysis parts of the study, and its success is largely predicated on correct model specification for the *exposure*. In contrast, the standardization G-Computation methods directly fit the outcome model and their success depends on correct model specification for the *outcome.* It is important to note that even in the presence of an appropriately formulated DAG (as discussed in Point 7) and appropriately selected set of covariates for adjustment (as demonstrated in Point 9), it is not guaranteed that a single a priori correct model for the outcome given exposure and covariates can be specified by the investigators. A number of different regression models each representing what may be reasonably expected with varying degrees of complexity (e.g. including various interaction terms) should be considered for G-Computation. The conditioning methods such as outcome regression, stratification, and matching require the correct model specification for the outcome as well as relying on the assumption of causal effect being the same within all confounder substrata. At present there is no consensus in the literature as to whether, and under what circumstances, a specific analytical method should be preferred.

While an in-depth discussion of quantitative bias analysis^23^ is beyond the scope of this primer, extensive sensitivity analyses that assess the robustness of the results to potential unmeasured or uncontrolled confounding are recommended as discussed by, for example, VanderWeele and Ding.^17^

Finally, a short comment on power analysis for a causal inference study based on routinely collected observational data. Unlike studies with prospective data collection, where the amount of data collected to ensure certain level of statistical power or estimate precision can be achieved by recruiting more participants, in research studies that use routinely collected health data without specific a priori research goals, the database population is intrinsically different to the study population, and only some (a priori unknown) portion of data may be relevant for answering a causal question. The discussions in Points 3, 6, 7, and 9 demonstrate that truly meaningful sample size estimation is impossible in practice. The currently recommended approach is to:

- formulate and analyze the causal question on observational data;
- present the estimates of the causal effect of interest with relevant measures of uncertainty in the form of confidence intervals, and
- once a sufficient number of estimates from diverse databases is available, meta-analyze the findings

It is important to stress that the emphasis in reporting should be placed on the magnitudes of the causal effects of interest and relevant measures of uncertainty and precision. Results that do not reach a chosen threshold of statistical significance should not be interpreted as the absence of the causal effect of interest, but rather as the absence of sufficient evidence for such an effect. This may be viewed rather like a neutral result from an underpowered clinical trial.

### Recommendation 10.1

The first step is to identify covariates to minimize the bias, using appropriate methods. Thereafter, adjust either by conditioning or by standardization logic. Use appropriate analytical methods to fit models for the exposure (i.e. propensity scores) or for the outcome (i.e. regression, matching, stratification or G-Computation).

### Recommendation 10.2

Avoid reporting “mutually adjusted” coefficients for all covariates included in the causal analysis regression models, since this may lead to “Table 2 fallacy.”

## References

[1] Benchimol EI Smeeth L Guttmann A , et al. The REporting of studies conducted using observational routinely-collected health data (RECORD) statement. PLoS Med 2015; 12: 12. 10.1371/journal.pmed.1001885 PMC4595218 26440803

[2] Vandenbroucke JP von Elm E Altman DG , et al. Strengthening the reporting of observational studies in Epidemiology (STROBE): Explanation and elaboration. PLoS Med 2007; 4: e297. 10.1371/journal.pmed.0040297 PMC2020496 17941715

[3] von Elm E Altman DG Egger M , et al. The strengthening the reporting of observational studies in Epidemiology (STROBE) statement: guidelines for reporting observational studies. PLoS Med 2007; 4: e296. 10.1371/journal.pmed.0040296 PMC2020495 17941714

[4] Garcia-Esperon C Bivard A Johns H , et al. Association of endovascular thrombectomy with functional outcome in patients with acute stroke with a large ischemic core. Neurology 2022; 99: e1345–e1355. 10.1212/WNL.0000000000200908 35803723

[5] Fox MP Murray EJ Lesko CR , et al. On the need to revitalize descriptive epidemiology. Am J Epidemiol 2022; 191: 1174–1179. 35325036 10.1093/aje/kwac056 PMC9383568

[bibr6-23969873251332118] Conroy S Murray EJ Conroy S. Let the question determine the methods: descriptive epidemiology done right. Br J Cancer 2020; 123: 1351–1352. 32814836 10.1038/s41416-020-1019-z PMC7592039

[bibr7-23969873251332118] Hernán MA. The C-Word: scientific euphemisms do not improve causal inference from observational data. Am J Public Health 2018; 108: 616–619. 29565659 10.2105/AJPH.2018.304337 PMC5888052

[bibr8-23969873251332118] Hernán MA Hsu J Healy B. A second chance to get causal inference right: a classification of data science tasks. Chance 2019; 32: 42–49.

[9] de Havenon A Sheth KN Johnston KC , et al. Effect of adjusting for baseline stroke severity in the national inpatient sample. Stroke 2021; 52: e739–e741. 10.1161/STROKEAHA.121.035112 PMC8545762 34455821

[10] Porta M . (ed.). A dictionary of epidemiology. 6th ed. New York City, NY: Oxford University Press, 2016.

[11] Rubin DB. Estimating causal effects of treatments in randomized and nonrandomized studies. J Educ Psychol 1974; 66: 688–701.

[bibr12-23969873251332118] Rubin DB. Inference and missing data. Biometrika 1976; 63: 581.

[bibr13-23969873251332118] Rubin DB. Assignment to treatment group on the basis of a covariate. J Educ Stat 1977; 2: 1–26.

[bibr14-23969873251332118] Rubin DB. Randomization analysis of experimental data: the Fisher randomization test comment. J Am Stat Assoc 1980; 75: 591–593.

[15] Rubin DB. Comment: which ifs have causal answers. J Am Stat Assoc 1986; 81: 961. DOI: 10.1080/01621459.1986.10478355

[16] Westreich D Cole SR. Invited commentary: positivity in practice. Am J Epidemiol 2010; 171: 674–677. 20139125 10.1093/aje/kwp436 PMC2877454

[17] VanderWeele TJ Ding P. Sensitivity analysis in observational research: introducing the e-value. Ann Intern Med 2017; 167: 268–274. 28693043 10.7326/M16-2607

[18] Austin PC. Different measures of treatment effect for different research questions. J Clin Epidemiol 2010; 63: 9–10. 19837563 10.1016/j.jclinepi.2009.07.006

[19] Lash TL Fox MP MacLehose RF , et al. Good practices for quantitative bias analysis. Int J Epidemiol 2014; 43: 1969–1985. 25080530 10.1093/ije/dyu149

[20] Merriam-Webster. Bias, https://www.merriam-webster.com/dictionary/bias (accessed 1 December 2024).

[21] Digitale JC Martin JN Glymour MM . Tutorial on directed acyclic graphs. J Clin Epidemiol 2022; 142: 264–267. 34371103 10.1016/j.jclinepi.2021.08.001 PMC8821727

[22] Hernán MA Sauer BC Hernández-Díaz S , et al. Specifying a target trial prevents immortal time bias and other self-inflicted injuries in observational analyses. J Clin Epidemiol 2016; 79: 70–75. 27237061 10.1016/j.jclinepi.2016.04.014 PMC5124536

[23] Brown JP Hunnicutt JN Ali MS , et al. Quantifying possible bias in clinical and epidemiological studies with quantitative bias analysis: common approaches and limitations. BMJ 2024; 385: e076365. 10.1136/bmj-2023-076365 38565248

[24] Hernán MA Alonso A Logan R , et al. Observational studies analyzed like randomized experiments: an application to postmenopausal hormone therapy and coronary heart disease. OA Epidemiol 2008; 19: 766–779. 10.1097/EDE.0b013e3181875e61 PMC3731075 18854702

[bibr25-23969873251332118] Hernán MA Robins JM. Using big data to emulate a target trial when a randomized trial is not available. Am J Epidemiol 2016; 183: 758–764. 26994063 10.1093/aje/kwv254 PMC4832051

[26] Moreno-Betancur M. The target trial: a powerful device beyond well-defined interventions. OA Epidemiol 2021; 32: 291–294. 10.1097/EDE.0000000000001318 33323746

[27] Andrew NE Ung D Olaiya MT , et al. The population effect of a national policy to incentivize chronic disease management in primary care in stroke: a population-based cohort study using an emulated target trial approach. The Lancet Regional Health – Western Pacific 2023; 34: 100723. 37283975 10.1016/j.lanwpc.2023.100723 PMC10240379

[28] Franklin JM Patorno E Desai RJ , et al. Emulating randomized clinical trials with nonrandomized real-world evidence studies. Circulation 2021; 143: 1002–1013. 33327727 10.1161/CIRCULATIONAHA.120.051718 PMC7940583

[29] VanderWeele TJ Robins JM. Four types of effect modification: a classification based on directed acyclic graphs. OA Epidemiol 2007; 18: 561–568. 10.1097/EDE.0b013e318127181b 17700242

[30] Textor J van der Zander B Gilthorpe MS , et al. Robust causal inference using directed acyclic graphs: the R package ‘dagitty’. Int J Epidemiol 2017; 45: 1887–1894. 10.1093/ije/dyw341 28089956

[31] Weiss NS. Clinical epidemiology: the study of the outcome of illness. 2nd ed. New York City, NY: Oxford University Press, 2006.

[32] Kaufman JS. Statistics, adjusted statistics, and maladjusted statistics. Am J Law Med 2017; 43: 193–208. 29254468 10.1177/0098858817723659

[33] Westreich D Greenland S. The Table 2 fallacy: presenting and interpreting confounder and modifier coefficients. Am J Epidemiol 2013; 177: 292–298. 23371353 10.1093/aje/kws412 PMC3626058

[34] Fekadu G Chelkeba L Kebede A. Risk factors, clinical presentations and predictors of stroke among adult patients admitted to stroke unit of Jimma university medical center, south west Ethiopia: prospective observational study. BMC Neurol 2019; 19: 327. 31847818 10.1186/s12883-019-1564-3 PMC6916064

[35] VanderWeele TJ VanderWeele TJ. Principles of confounder selection. Eur J Epidemiol 2019; 34: 211–219. 30840181 10.1007/s10654-019-00494-6 PMC6447501

[36] Robins J. A new approach to causal inference in mortality studies with a sustained exposure period—application to control of the healthy worker survivor effect. Math Model 1986; 7: 1393–1512.

[37] Chatton A Rohrer JM. The causal cookbook: recipes for propensity scores, G-Computation, and doubly robust standardization. Advances in Methods and Practices in Psychological Science 2024; 7: 25152459241236149.

[38] Thomas LE Li F Pencina MJ. Overlap weighting: a propensity score method that mimics attributes of a randomized clinical trial. JAMA 2020; 323: 2417–2418. 32369102 10.1001/jama.2020.7819

[39] Austin PC Yu AYX Vyas MV , et al. Applying propensity score methods in clinical research in neurology. Neurology 2021; 97: 856–863. 34504033 10.1212/WNL.0000000000012777 PMC8610625

